# A female with rash and facial swelling

**DOI:** 10.4103/0974-2700.62121

**Published:** 2010

**Authors:** Audrey Tan, Michael B Stone

**Affiliations:** Department of Emergency Medicine, SUNY Downstate/Kings County Hospital Center, Brooklyn, NY 11203, USA

A 34-year-old female presented to the emergency room (ER) with a diffuse rash and facial swelling. The patient was 5 weeks postpartum and had been prescribed phenytoin after she had experienced seizures shortly after delivery. Two weeks after starting phenytoin, the patient developed a diffuse erythematous rash. Phenytoin was therefore discontinued and instead levetiracetam was initiated. The patient came to the ER as her symptoms had continued to progress. Examination revealed the following; temperature 100.5°F, pulse rate 107/min, blood pressure 115/76 mm Hg, and respiratory rate 18/min; pulse oximetry showed 100% oxygenation while breathing room air. Physical exam demonstrated facial edema; ulcerative lesions on the inner lip, buccal mucosa, and roof of the mouth; [Figure 1] and a diffuse morbilliform rash on the extremities, chest, and back [Figure 2]. Laboratory analysis demonstrated a total white blood cell count of 26,300 K per microliter; the differential count showed 22% eosinophils. The ALT was 135 U/L and the AST 61 U/L. The patient was admitted to the hospital for systemic steroid therapy and intravenous fluid administration.

**Figure 1:**
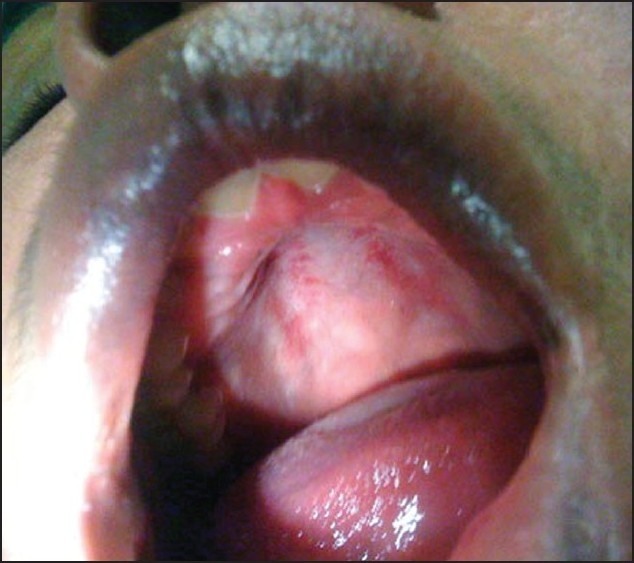
Ulcerative lesions visualized on the roof of the mouth

**Figure 2:**
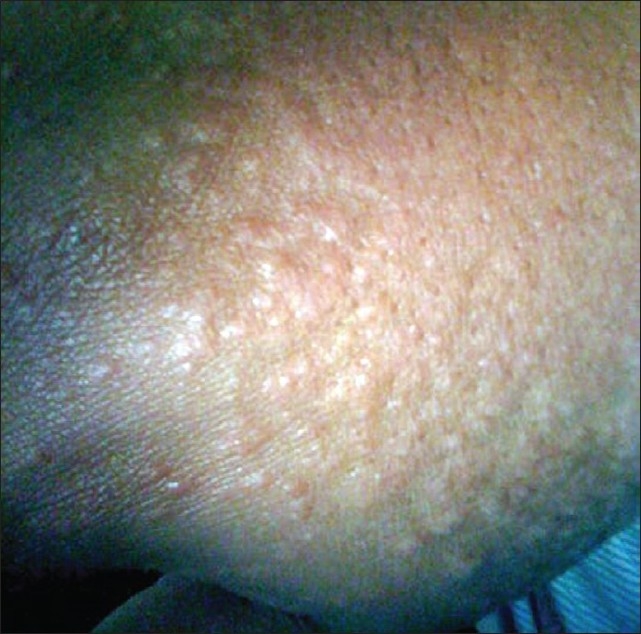
Morbilliform rash on the patient's forearm

## DISCUSSION

Diagnosis: Drug reaction with eosinophilia and systemic symptoms (DRESS)

Drug reaction with eosinophilia and systemic symptoms (DRESS) is a hypersensitivity reaction that develops 1–8 weeks after medication exposure and is characterized by persistent fever, marked eosinophilia, hepatic dysfunction, and facial edema, with pinpoint pustules and a diffuse morbilliform rash that can progress to vesiculation, bullae formation, and erythroderma.[1,2] The most commonly implicated medications are sulfonamides, anticonvulsants (e.g., phenobarbital, carbamazepine, phenytoin), sulfasalazine, dapsone, and allopurinol. The mortality of DRESS syndrome is 10% and is mostly due to the occurrence of visceral complications, including fulminant hepatitis, interstitial nephropathy, eosinophilic interstitial pneumopathy, pericarditis, myocarditis, or pancreatitis.[3] Treatment of DRESS includes withdrawal of the drug and administration of systemic steroids for 4–6 weeks; this may prevent relapse and/or progression of systemic involvement. Use of both intravenous immunoglobulin and plasmapheresis has been described but is not yet considered standard care. Following discharge, close monitoring of disease resolution (including renal, thyroid and, hepatic function) is necessary.[2]

## CONCLUSIONS

1. Drug reaction with eosinophilia and systemic symptoms (DRESS) is a hypersensitivity reaction to a drug; it is characterized by fever, eosinophilia, hepatic dysfunction, facial edema, and a diffuse rash.
2. Sulfonamides, anticonvulsants, sulfasalazine, dapsone, and allopurinol are the most commonly implicated medications.
3. There is 10% mortality. Treatment involves withdrawal of the drug and administration of a 4–6 week course of systemic steroids.
